# The Reactions of H_2_O_2_ and GSNO with the Zinc Finger Motif of XPA. Not A Regulatory Mechanism, But No Synergy with Cadmium Toxicity

**DOI:** 10.3390/molecules25184177

**Published:** 2020-09-12

**Authors:** Aleksandra Witkiewicz-Kucharczyk, Wojciech Goch, Jacek Olędzki, Andrea Hartwig, Wojciech Bal

**Affiliations:** 1 Institute of Biochemistry and Biophysics, Polish Academy of Sciences, Pawińskiego 5a, 02-106 Warsaw, Poland; alwitk@gmail.com (A.W.-K.); jaole@ibb.waw.pl (J.O.); 2 Faculty of Pharmacy, Medical University of Warsaw, Banacha 1, 02097 Warsaw, Poland; wojciech_goch@wp.pl; 3 Institute of Applied Biosciences, Karlsruhe Institute of Technology, 76021 Karlsruhe, Germany; andrea.hartwig@kit.edu

**Keywords:** zinc fingers, zinc, cadmium, hydrogen peroxide, S-nitrosoglutathione, oxidation

## Abstract

Tetrathiolate zinc fingers are potential targets of oxidative assault under cellular stress conditions. We used the synthetic 37-residue peptide representing the tetrathiolate zinc finger domain of the DNA repair protein XPA, acetyl-DYVICEECGKEFMSYLMNHFDLPTCDNCRDADDKHK-amide (XPAzf) as a working model to study the reaction of its Zn(II) complex (ZnXPAzf) with hydrogen peroxide and S-nitrosoglutathione (GSNO), as oxidative and nitrosative stress agents, respectively. We also used the Cd(II) substituted XPAzf (CdXPAzf) to assess the situation of cadmium assault, which is accompanied by oxidative stress. Using electrospray mass spectrometry (ESI-MS), HPLC, and UV-vis and circular dichroism spectroscopies we demonstrated that even very low levels of H_2_O_2_ and GSNO invariably cause irreversible thiol oxidation and concomitant Zn(II) release from ZnXPAzf. In contrast, CdXPAzf was more resistant to oxidation, demonstrating the absence of synergy between cadmium and oxidative stresses. Our results indicate that GSNO cannot act as a reversible modifier of XPA, and rather has a deleterious effect on DNA repair.

## 1. Introduction

The zinc fingers (ZF) comprise one of the most abundant and diverse motifs in protein biochemistry, well beyond the classical structures in which the Zn(II) ion bound to two Cys and two His residues serves as a tetrahedral pin to create a DNA-recognizing loop between a β-sheet and an α-helix [1]. The object of our interest, XPAzf, is a nonclassical four-Cys ZF present in the 273aa XPA nuclear protein belonging to the Nucleotide Excision Repair (NER) DNA repair pathway [2]. The function of XPA is to support the full opening of the DNA lesion, together with XPG and RPA DNA binding factors, and XPAzf participates in the formation of the multiprotein repair complex [2]. More recently XPA was proposed to act also as processivity factor, mediating the functions of XPF and XPG proteins [3]. XPA is a necessary NER component, as evidenced by Xeroderma Pigmentosum type A, a severe genetic disorder characterized with the impairment of DNA repair with consequences in UV hypersensitivity and increased cancer frequency [4]. The function of XPA is lost in this condition due to mutations. NER can also be inhibited by chemical carcinogens targeting XPA, including Ni(II), Co(II) and Cd(II) ions, and selenium and arsenic compounds [5,6,7]. In our previous studies we demonstrated that a direct substitution of Zn(II) in ZnXPAzf by any of these metal ions is mechanistically possible and leads to structural alterations which are likely responsible for this inhibition [8,9]. While Ni(II) and Co(II) substitutions increased the XPAzf susceptibility to oxidation, Cd(II) substitution decreased it. Cd(II) was also the only metal ion with the binding affinity higher than that of Zn(II) (*K*_d_ of 0.2 pM vs. 0.16 nM at pH 7.4) [8,9]. This type of behavior is common for tetrathiolate ZFs [10].

H_2_O_2_ is a major reactive oxygen species (ROS) byproduct of oxygen metabolism, present intracellularly at a basal concentrations about 10 nM [11]. Being uncharged and non-radical, it can diffuse to long distances and penetrates membranes. It has a number of physiological functions as mildly reactive redox messenger, but elevated during oxidative stress it is disruptive to cellular metabolism. We demonstrated that it can destroy ZnXPAzf at a 10-fold molar excess by gradual formation of disulfide bonds and Zn(II) expulsion [12].

Nitric oxide is a very important second messenger in the human body both extra- and intracellularly [13]. The NO molecule acts largely extracellularly, controlling a number of systemic function, such as blood pressure via vasodilation. However, despite its relatively controllable reactivity as for a radical species, its message inside cells is largely conveyed by exchange mechanisms between the targeted protein thiols and low molecular carriers, predominantly S-nitrosoglutathione (GSNO) [14]. Generally a physiological effector, GSNO can also be part of nitrosative stress when abnormally elevated [15].

In our previous study we demonstrated that ZnXPAzf can be inactivated by GSNO at a 10-fold molar excess [16]. The molecular mechanism of this reaction included S-nitrosylation, S-glutathionylation and intramolecule disulphide bonds formation. The GSNO complexation to Zn(II) ion in ZnXPAzf was proposed to be an initial step of this process. In early stages of the reaction we also observed nitrosylation of a single thiolate without the Zn(II) release. Such potentially reversible reaction stages could act as regulators of XPA activity. In this study we attempted to verify this idea using lower GSNO concentrations and H_2_O_2_ as comparator. We also investigated the behavior of Cd(II)-substituted XPAzf in analogous conditions, in order to find out whether there could be synergy between the Cd(II) toxicity (known to elicit intracellular oxidative stress on its own [17]) and H_2_O_2_ and GSNO, as oxidative stress effectors. We used synthetic acetyl-DYVICEECGKEFMSYLMNHFDLPTCDNCRDADDKHK-amide as XPAzf model, as in our previous work [6,7,8,9,12,16].

## 2. Experimental Procedures

*Sample preparation and measurements*. All XPAzf samples were prepared under reduced oxygen (Coy Glove Box, O_2_ level <0.5%). The XPAzf concentration was verified spectrophotometrically and with the use of standard DTNB assay [18]. Ten micrometer XPAzf samples in the presence of equimolar Zn(II) or Cd(II), added from sulfate stock solutions, were exposed to equimolar, and two- and three-fold excess of H_2_O_2_ or GSNO, prepared as published previously [12,16]. The 10 mM ammonium acetate and sodium phosphate buffers, pH 7.4 were prepared, degassed and stored in the glove box. The samples for spectroscopic experiments were sealed in quartz cuvettes before withdrawal to ambient atmosphere. The control samples of metal-free XPAzf were prepared analogously. All experiments were performed at 25 °C. Reaction samples were withdrawn periodically and separated by HPLC (Waters Breeze) on a C18 column, as described [12]. The peaks were collected and analyzed using a Q-Tof Premier ESI-MS instrument from Waters, using the following parameters: Capillary 3.1 kV, sampling cone 66 V, extraction cone 2 V, source temperature 80 °C, ion guide 3.9 V, desolvatation temperature 40 °C. XPAzf reaction products were best detectable for the 4+ ion which was selected for quantitative analysis. Circular Dichroism spectra were obtained on a JASCO J-815 spectropolarimeter, using d = 5 mm quartz cuvettes over the spectral range of 190–280 nm. UV-Vis monitored Zn(II) release according to the 4-(2-Pyridylazo)-resorcinol monosodium salt (PAR) assay was performed as published [19], using either a Cary 50 Bio or a Perkin Elmer Lambda 25 spectrometer and d = 10 mm cuvettes. The data obtained from HPLC signal integration were used in calculations of 2nd order rates of the holo-ZF decay, using Mathematica 9 software package.

## 3. Results and Discussion

**Figure 1:**
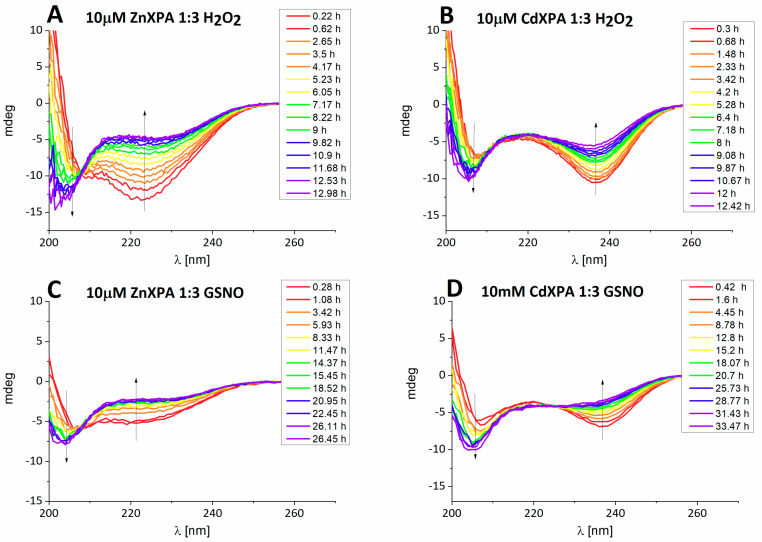
Examples of the time course of 10 μM ZnXPAzf and CdXPAzf decomposition by 3-fold molar excess of H_2_O_2_ or GSNO in 10 mM ammonium acetate buffer, pH 7.4, followed by CD spectroscopy. (**A**) ZnXPAzf and H_2_O_2_; (**B**) CdXPAzf and H_2_O_2_; (**C**) ZnXPAzf and GSNO; (**D**) CdXPAzf and GSNO. The time points (hours) are indicated in the graph.

Kinetic studies. The reactions were followed by CD, HPLC and the PAR Zn(II) release assay, with very consistent results. The CD spectrum of ZnXPAzf was characterized with a strong negative band at 223 nm, originating from the Zn-S charge transfer (CT) band. The analogous Cd-S CT band was present at 237 nm for CdXPAzf [9]. Figure 1 presents the representative reactions, obtained for three-fold excess of either oxidizing agent, performed at pH 7.4 (10 mM ammonium acetate). In all cases a gradual loss of metal ion was observed.

**Figure 2:**
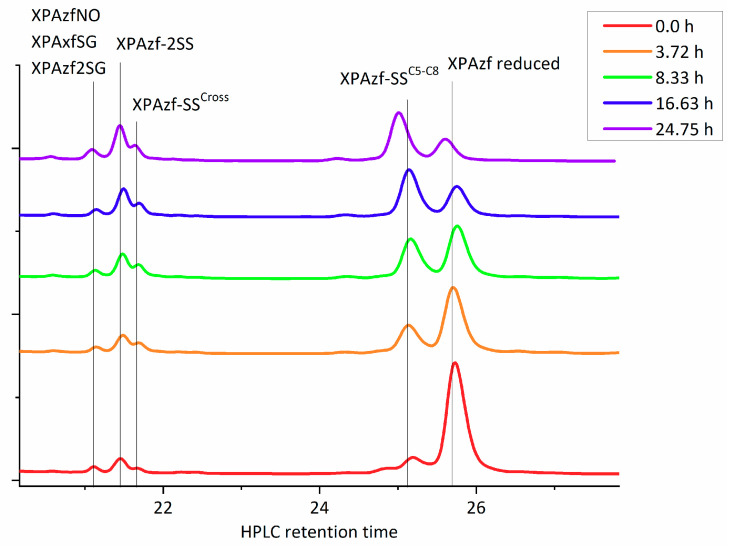
The exemplary set of HPLC chromatograms obtained for the reaction of 10 μM ZnXPAzf with a three-fold molar excess of S-nitrosoglutathione (GSNO). The times of the reaction [h] and peak assignments are given in the graph. The label SS^C5-C8^ indicates the presence of C5-C8 disulfide bond, the label SS^cross^ denotes the presence of disulfide bridges involving either C5 or C8 on one side and either C26 or C29 on another. The assignments are based on previous results [12] and the ESI-MS analysis.

The analytical HPLC separations were performed under acidic conditions (0.1% TFA, pH~2), resulting in the metal ion loss during sample preincubation with the running buffer. Therefore, only the covalent reaction products were observed. However, as demonstrated previously, no peptide oxidation occurred during the HPLC analysis [12,16]. Furthermore, due to high affinity constants for Zn(II) and Cd(II) complexes of XPAzf [8,9] all peptide was fully metallated in each experiment prior to incubation with oxidants and the loss of metal was only caused by peptide oxidation. Therefore, the reduced apopeptide in the HPLC analysis represented quantitatively the metallated peptide in the reaction. Each HPLC peak was collected and its contents was immediately analyzed using ESI-MS. These chromatograms revealed the repeating pattern of the XPAzf forms, with the representative example given in Figure 2.

XPAzf contains two pairs of adjacent Cys residues: C5/C8 and C26/C29. Our previous study using thiol derivatization and MS fragmentation experiments indicated the preferential formation of the C5-C8 disulfide as the first oxidation product, sufficient to release the metal ion [12]. This was clearly confirmed in the current experiments (the XPAzf-1SS^C5-C8^ peak at ~25.1 min., identified by ESI-MS and retention time). Minor peaks at 21.7, 21.5, and 21.2 min. could be identified as XPAzf-1SS^cross^ (a mixture of products with single disulfide bonds other than C5-C8 or C26-C29, XPAzf-2SS and the mixture of XPAzf nitrosylated and glutathionylated species, respectively. The last peak appeared only in the reaction with GSNO.

**Figure 3:**
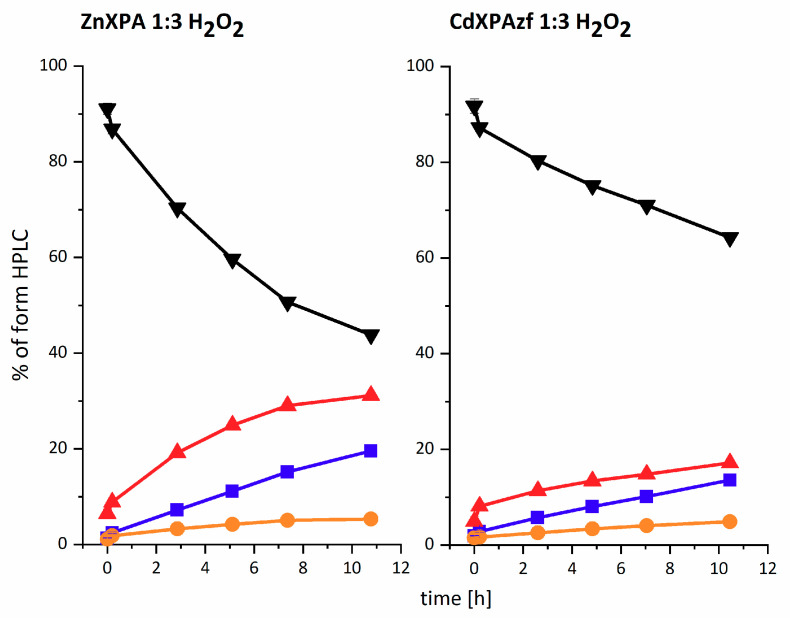
The kinetics of reactions of 10 μM ZnXPAzf and CdXPAzf with a three-fold excess of H_2_O_2_, plotted from the normalized HPLC peaks. Black—reduced XPAzf (in fact MeXPAzf complex), red—XPAzf-SS^C5-C8^, blue—XPAzf-SS^cross^, orange—XPAzf-2SS.

**Scheme 1:**
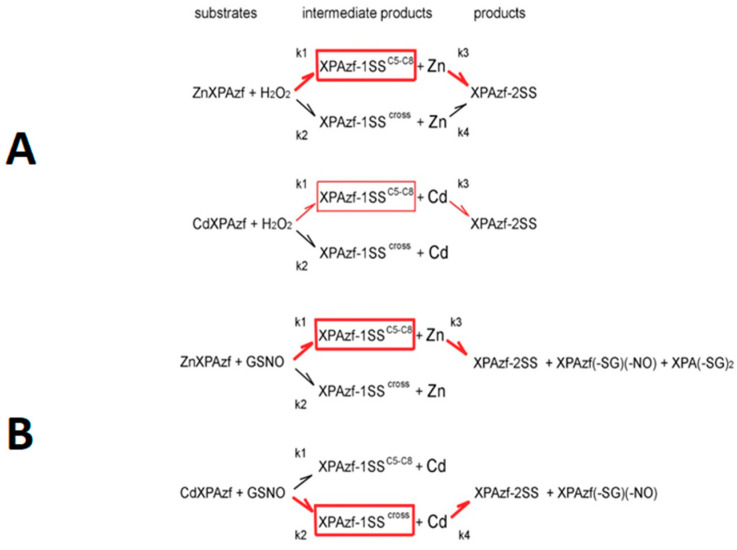
The mechanism of ZnXPAzf and CdXPAzf reaction with H_2_O_2_ (**A**) and GSNO (**B**) The size of the arrows presents the major reaction pathways, and the frames the major intermediate products, common for all three oxidant concentrations.

**Scheme 2:**
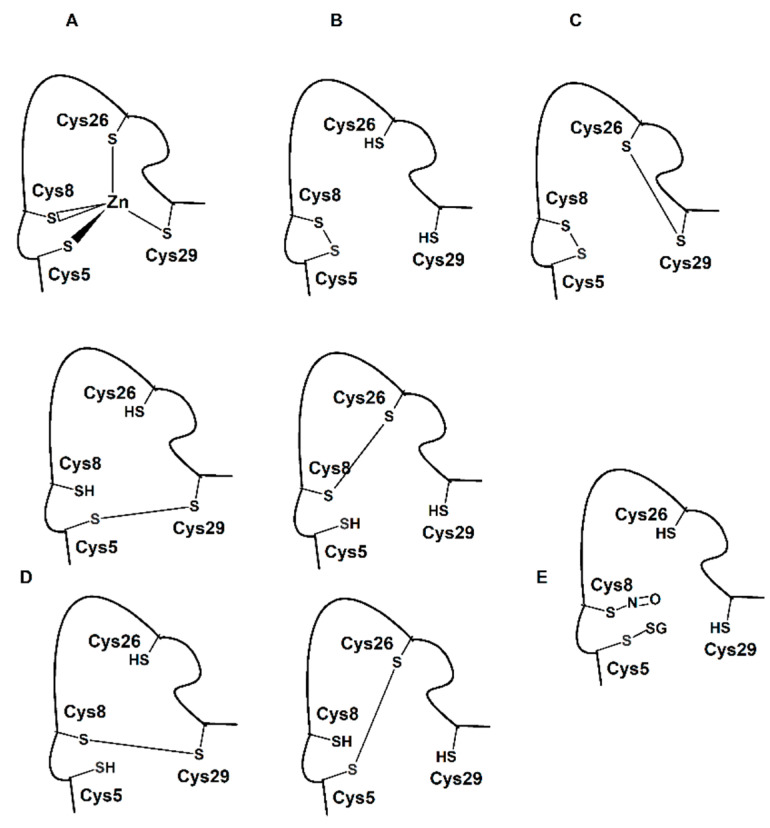
The key reaction products of ZnXPAzf reactions with H_2_O_2_ and GSNO. (**A**) ZnXPAzf; (**B**) XPAzf-1SS^C5-C8^; (**C**) XPAzf-2SS; (**D**) four variants of XPAzf-1SS^cross^; (**E**), example of XPAzf(-SG)(-NO). The products of CdXPAzf reactions are analogous.

Reaction mechanism, H_2_O_2_. The comparison of information provided by all three methods employed in kinetic studies indicated that the formation of a single disulfide bond was sufficient to actuate the metal ion release not only for ZnXPAzf, as seen previously [12,16] but also for CdXPAzf, despite the much higher affinity of Cd(II) to thiolate ligands [9,10]. All data consistently show that the C5/C8 pair is most susceptible to oxidation for both metal ions. The final double disulfide product was always detected as a single HPLC peak which suggests that it contains the C5-C8 and C26-C29 bridges. This would require a disulfide reshuffling in the XPAzf–SS^cross^, explaining the relative persistence of this minor species. The time course for these reactions is illustrated in Figure 3 for the three-fold molar excess of H_2_O_2_, with the trend maintained for all three H_2_O_2_ concentrations (see Table 1 below). The data for all three H_2_O_2_ concentrations were pooled together and fed into the reaction model shown at the top of Scheme 1. The resulting rate constants are presented in Table 1. The proposed structures of reaction products are presented in Scheme 2A–D.

Comparing the rates, one can note that the preference of the k_1_ mechanism over k_2_ for the first disulfide formation in ZnXPAzf is much stronger than in CdXPAzf. We interpret it as evidence for alteration of spatial positions of Cys residues in the larger CdS_4_ core compare to the ZnS_4_ core. The reaction is overall slower for CdXPAzf, but not by as much as would be expected on the basis of the affinity difference [9]. Apparently the enthalpic stabilization provided by Cd(II) is partially overcome by the better oxidant accessibility to all sulfurs, not just C5/C8 in CdXPAzf. The k_3_ values are the same. This is expected, because this process involves the demetalated ZF peptide. The absence of k_4_ for the Cd(II) case is probably due to the slower reaction and insufficient accumulation of the relevant intermediates during 12 h incubations.

**Figure 4:**
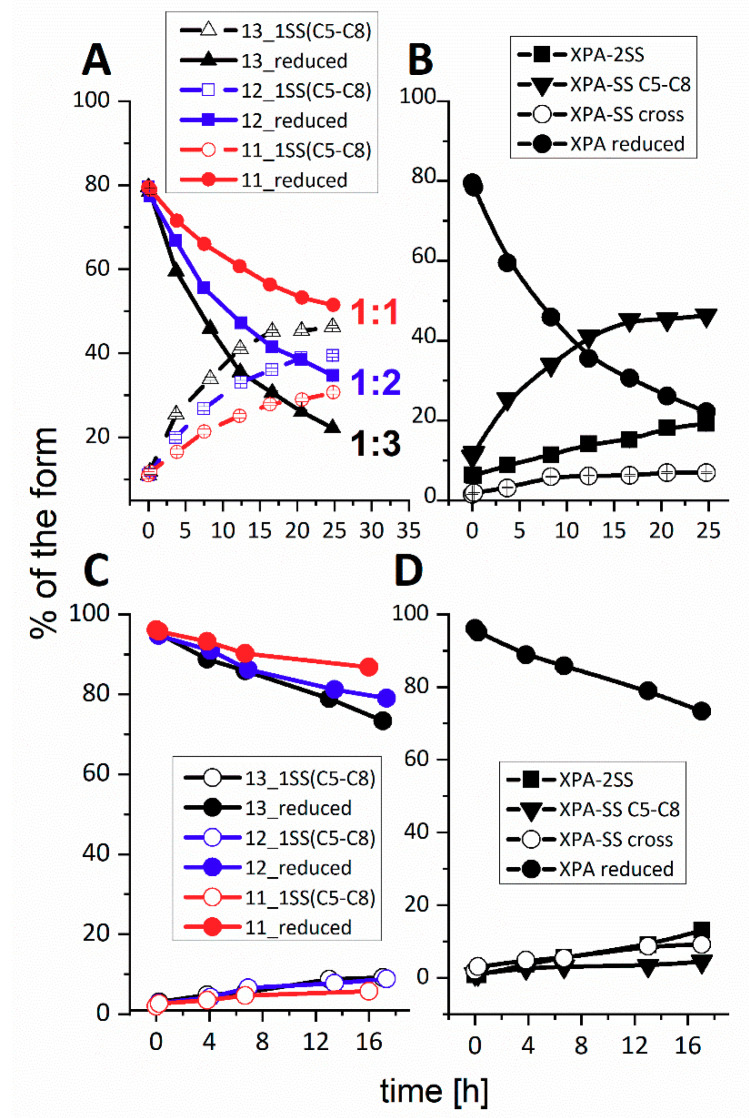
(**A**) The normalized areas of the XPAzf peaks obtained from HPLC after the ZnXPAzf reaction with GSNO and examined with ESI-MS (GSNO concentrations are color coded on the graph). Solid lines trace the reduced XPAzf, which represents the ZnXPAzf complex, dashed lines trace the intermediate XPA-SS^C5-C8^. (**B**) Formation of disulfide products for 10 μM ZnXPAzf and a three-fold excess of GSNO. (**C**) The normalized areas of the XPAzf peaks obtained from HPLC after the CdXPAzf reaction with GSNO and examined with ESI-MS (GSNO concentrations are color coded on the graph). Solid lines trace the reduced XPAzf, which represents the CdXPAzf complex, dashed lines trace the intermediate XPA-SS^C5-C8^. (**D**) Formation of disulfide products for 10 μM CdXPAzf and a three-fold excess of GSNO.

Reaction mechanism, GSNO. In GSNO reactions with ZnXPAzf we observed the formation of the four types of products. The pattern of the intermediate and final products was similar to that observed with H_2_O_2_, with a strong preference of the initial formation of XPA-SS^C5-C8^, with a small addition (about 4%) of the nitrosylated/glutathionylated (XPAzf-SG-NO), glutathionylated (XPAzf-SG) and glutathionylated (XPAzf-2SG) species, all contained in one HPLC peak. The presence of these forms was confirmed by ESI-MS analysis of HPLC fractions. In the reaction of CdXPAzf with GSNO, however, there was a surprising preference for the formation of XPAzf-SS^cross^. The double glutathionylated forms of XPAzf (XPAzf-2SG), not seen for the Zn(II) complex, appeared at a very low level. Nevertheless, XPAzf-2SS was the final product in this reaction as well. In Figure 4 one can see that the deterioration of CdXPAzf complex was much slower than that of ZnXPAzf.

The mechanism of GSNO reaction for ZnXPAzf, presented at the bottom of Scheme 1 is very similar to that for H_2_O_2_. The only difference is the absence of the k_4_ process, apparently due to the insufficient accumulation of XPAzf-SS^cross^ in a slower reaction, similarly to that for CdXPAzf and H_2_O_2_. A more significant difference is present for the reaction of CdXPAzf with GSNO, where the weights of the k_1_/k_3_ and k_2_/k_4_ pathways was reversed. The structures of reaction products are presented in Scheme 2.

The rate constant values presented in Table 1 and Table 2 allow us to observe that at low exposures H_2_O_2_ and GSNO can oxidize ZnXPAzf and CdXPAzf with similar efficacies, limited by the character of the metal ion, rather than the oxidant. The individual k_1_ and k_2_ values indicate that GSNO is slightly less selective than H_2_O_2_ in preferential targeting of the C5 and C8 cysteines, but the analogous effect of Cd(II) substitution is stronger. Nevertheless, Cd(II) protects the XPAzf thiols much more effectively than Zn(II).

Biological significance. Our chemical study indicated that both H_2_O_2_ and GSNO oxidize ZnXPAzf with the release of Zn(II) ions, with similar reaction rates. The process is slow, on the timescale of hours to days, but is irreversible, with no accumulation of a quasi-reversible intermediate. The presence of such for GSNO was hinted by our previous research performed at a higher GSNO concentration [16]. However, the current study indicates that S-nitrosylated species observed previously were formed as byproducts of a quicker reaction enforced by a high GSNO excess rather than constituting the main reaction pathway. Therefore, we can suggest that GSNO is potentially deleterious against ZnXPAzf and possibly any other tetrathiolate ZFs, with the only protection provided by the sluggishness of the reaction. We can therefore propose that transnitrosylation by GSNO is strongly unlikely to be a regulatory signal for ZF proteins. On the other hand, Cd(II) substitution conferred higher resistance to the oxidative assault by both agents, thus indicating that the oxidative/nitrosative stress is not necessarily acting synergistically with cadmium toxicity.

## Figures and Tables

**Table 1:** Logarithmic values for 2nd order rate constants (M^−2^·h^−1^) obtained for reactions of ZnXPAzf and CdXPAzf with H_2_O_2_. The k_1_, k_2_, k_3_, and k_4_ pathways are defined in Scheme 1.

	Log k_1_	Log k_2_	Log k_3_	Log k_4_
ZnXPAzf	4.64 ± 0.04	3.75 ± 0.24	4.52 ± 0.07	4.16 ± 0.45
CdXPAzf	3.26 ± 0.11	3.12 ± 0.14	4.58 ± 0.14	<2.0

**Table 2:** Logarithmic values for 2nd order rate constants (M^−2^·h^−1^) obtained reactions of ZnXPAzf and CdXPAzf with GSNO. The k_1_, k_2_, k_3_, and k_4_ pathways are defined in Scheme 2.

	Log k_1_	Log k_2_	Log k_3_	Log k_4_
ZnXPAzf	4.51 ± 0.03	4.12 ± 0.08	4.92 ± 0.09	<2.0
CdXPAzf	3.31 ± 0.19	3.49 ± 0.14	<2.0	4.78 ± 0.14
